# Variation in fine-scale recombination rate in temperature-evolved *Drosophila melanogaster* populations in response to selection

**DOI:** 10.1093/g3journal/jkac208

**Published:** 2022-08-12

**Authors:** Ari Winbush, Nadia D Singh

**Affiliations:** Department of Biology, Institute of Ecology and Evolution, University of Oregon, Eugene, OR 97403, USA; Department of Biology, Institute of Ecology and Evolution, University of Oregon, Eugene, OR 97403, USA

**Keywords:** *Drosophila melanogaster*, fine-scale recombination rate, experimental evolution, temperature

## Abstract

Meiotic recombination plays a critical evolutionary role in maintaining fitness in response to selective pressures due to changing environments. Variation in recombination rate has been observed amongst and between species and populations and within genomes across numerous taxa. Studies have demonstrated a link between changes in recombination rate and selection, but the extent to which fine-scale recombination rate varies between evolved populations during the evolutionary period in response to selection is under active research. Here, we utilize a set of 3 temperature-evolved *Drosophila melanogaster* populations that were shown to have diverged in several phenotypes, including recombination rate, based on the temperature regime in which they evolved. Using whole-genome sequencing data from these populations, we generated linkage disequilibrium-based fine-scale recombination maps for each population. With these maps, we compare recombination rates and patterns among the 3 populations and show that they have diverged at fine scales but are conserved at broader scales. We further demonstrate a correlation between recombination rates and genomic variation in the 3 populations. Lastly, we show variation in localized regions of enhanced recombination rates, termed warm spots, between the populations with these warm spots and associated genes overlapping areas previously shown to have diverged in the 3 populations due to selection. These data support the existence of recombination modifiers in these populations which are subject to selection during evolutionary change.

## Introduction

Homologous recombination is a critical process in which genetic material is transferred between nucleic acid strands. During meiosis, this exchange occurs between homologous chromosomes and is essential for proper chromosomal segregation. In addition, recombination plays a critical evolutionary role primarily through the disruption of linkage disequilibrium (LD), creation of new haplotypes, and increasing genetic variation allowing for modifications to fitness (Hill and Robertson 1966; Begun and Aquadro 1992; Kliman and Hey 1993; Kong *et al.* 2004; Baudat *et al.* 2013). Likewise, the tight control of the recombination rate is important in maintaining fitness while preventing aneuploidy (Barton and Charlesworth 1998; Ritz *et al.* 2017; Stapley *et al.* 2017).

Given this important role, the recombination rate variation has been studied extensively and in numerous organisms. These studies have demonstrated almost universally the existence of recombination rate variation across genomes, within and among populations, as well as between sexes and species at both fine and broad scales (e.g. Nachman 2002; Crawford *et al.* 2004; Cirulli *et al.* 2007; Mancera *et al.* 2008; Dumont *et al.* 2009; Singh *et al.* 2009; Stevison and Noor 2010; Hinch *et al.* 2011; Smukowski and Noor 2011; Chan *et al.* 2012; Comeron *et al.* 2012; Manzano-Winkler *et al.* 2013; Singh *et al.* 2013; Singhal *et al.* 2015; Smukowski *et al.* 2015; Hunter *et al.* 2016; Stapley *et al.* 2017; Dreissig *et al.* 2019; Shanfelter *et al.* 2019; Apuli *et al.* 2020; Hasan and Ness 2020; Samuk *et al.* 2020). Noteworthy among studies of recombination rate are: (1) the conservation of recombination rate variation and landscapes at broad scales between closely related species and populations and (2) the rapid divergence and evolution of recombination rate variation at fine scales. The examples of the former include the variation in recombination rate observed around heterochromatic chromosomal structures and genomic motifs; for example, suppressed recombination rate around centromeres compared to other regions in fruit flies and yeast, or changes in recombination rate as a function of distance from telomeres in humans (Beadle 1932; Nachman 2002; Anderson *et al.* 2003; Chen *et al.* 2008; Myers *et al.* 2008; Comeron *et al.* 2012). Concerning the latter, the existence of localized recombination “hotspots” consisting of small regions in which the recombination rate is multifold higher than background has been observed in a variety of organisms. These hotspots appear to be relatively stable within populations yet vary significantly among species and between populations. These results suggest a rapid evolution of divergence at this scale (Myers *et al.* 2005; Ptak *et al.* 2005; Winckler *et al.* 2005; Mancera *et al.* 2008; Baudat *et al.* 2010; Smagulova *et al.* 2011; Baudat *et al.* 2013).

Another trend that has emerged from decades of work on recombination rate is that populations subjected to selection on a particular phenotype have been shown to also exhibit differences in recombination. This is seen in the context of both experimental evolution experiments and domestication. Concerning strong artificial selection due to domestication, studies have observed both changes in recombination rates, and polymorphisms in recombination-specific genes in domesticated plants and animals (Burt and Bell 1987; Charlesworth 1993; Ross-Ibarra 2004; Groenen *et al.* 2009; Poissant *et al.* 2010; Sidhu *et al.* 2017; Dreissig *et al.* 2019). Nevertheless, this phenomenon is not always observed in studies of domestication, and various underlying causes have been reported (Otto and Barton 2001; Muñoz-Fuentes *et al.* 2015). In the contexts of experimental evolution, the studies of natural populations that have adapted to local environments, and parasite–host coevolution, changes in recombination rate are also observed (Korol and Iliadi 1994; Korol 1999; Bourguet *et al.* 2003; Kerstes *et al.* 2012; Aggarwal *et al.* 2015, 2019; Neupane and Xu 2020).

Much study has therefore been devoted to elucidating the genetic basis and the evolution of fine-scale recombination rate variation. An important discovery in this area was the histone methyltransferase-encoding gene, *PRDM9*, shown in humans, mice, and other taxa to regulate the location of recombination hotspots (Oliver *et al.* 2009; Baudat *et al.* 2010; Berg *et al.* 2011; Hinch *et al.* 2011; Schwartz *et al.* 2014). However, many organisms lack *PRDM9*, and evidence would suggest additional genetic determinants are responsible for fine-scale recombination rate variation in these specimens.

*Drosophila* represents a commonly used model system for study of genetic determinants and evolution of fine-scale recombination rate variation. Lacking *PRDM9* and hotspots at the same scale as observed in humans and mice, numerous studies nevertheless have demonstrated recombination rate variation at fine scales among *Drosophila* species and within genomes (Cirulli *et al.* 2007; Singh *et al.* 2009; Stevison and Noor 2010; Chan *et al.* 2012; Comeron *et al.* 2012; Manzano-Winkler *et al.* 2013; Singh *et al.* 2013; Smukowski *et al.* 2015; Adrion *et al.* 2020). There is likewise evidence that selection acts on recombination rate both directly and indirectly. The latter occurs in the context of indirect selection of other traits in response to novel environments, experimental evolution, and artificial selection. This would suggest the existence of genes that act as “recombination modifiers” by affecting the recombination rate (Kidwell 1972; Charlesworth 1993; Korol and Iliadi 1994; Bourguet *et al.* 2003; Aggarwal *et al.* 2015; Smukowski *et al.* 2015). However, a comprehensive analysis of recombination rate variation across and within species and factors driving fine-scale recombination rate divergence remains lacking in *Drosophila*. For example, the existence of recombination modifiers would suggest that under conditions promoting rapid evolution and selection, areas of enhanced recombination would overlap genomic regions undergoing selection; however, the extent to which this occurs is unknown. The examination of fine-scale recombination rates in populations subject to selection during an experimental evolution period would provide insight into these questions.

We previously utilized a set of experimentally evolved *Drosophila melanogaster* populations that were subjected to one of 3 temperature treatments [Warm, Cold, and Temporally fluctuating (Temp)] over a 3-year period and were subsequently demonstrated to show fixed heritable differences in recombination rate between a set of visible markers (Kohl and Singh 2018). Those from the Warm and Cold regimes exhibited the highest and lowest rates, respectively, and the Temp lines exhibited an intermediate rate although no variation in recombination rate plasticity was observed (Kohl and Singh 2018). Whole-genome sequencing of these populations and subsequent analysis of single nucleotide polymorphism (SNP)-allele frequencies identified multiple regions with significant differences in SNP allele frequencies between the 3 populations stemming from pairwise comparisons (Warm vs Cold, Cold vs Temp, and Warm vs Temp; Winbush and Singh 2021). These regions of divergence overlapped regions of reduced nucleotide diversity and reduced empirical recombination rates, which suggest that these regions were subject to selection during the experimental evolution period. In addition, we were able to map divergent SNPs to potential candidate loci responsible for variation in recombination rate between the 3 populations (Winbush and Singh 2021). Interestingly, these loci showed overlap with candidate loci previously identified in a separate screen of 205 inbred lines of the *D. melanogaster* Genetic Reference Panel (DGRP) that investigated the genetic basis of variation in recombination rate at the population level. This suggested that candidate genes regulating recombination rate between populations and within populations might be shared (Hunter *et al.* 2016; Winbush and Singh 2021). Therefore, these 3 populations represent ideal candidates to test for both the divergence of recombination rate at fine scales in response to selection and potential overlap of localized areas of enhanced recombination rate with areas undergoing selection during the evolutionary period.

We took advantage of the existing LD-based statistical software, LDhelmet, to infer historical patterns of fine-scale recombination rate in these 3 populations. This offers advantages over empirical methods of assessing fine-scale recombination rates using pedigree analyses, which are often laborious requiring numerous controlled crosses and extensive genotyping. However, LD-based methods also have limitations due to other factors that influence patterns of LD across the genome. Like previous studies, we therefore compare our data to previously generated empirical data and show good correlation between the 2. This result is consistent with other studies comparing LDhelmet-generated data to empirical results (Singh *et al.* 2009; Chan *et al.* 2012; Smukowski *et al.* 2015) showing strong correlation and demonstrating the efficacy of this LD-based method (see *Results*).

Our LDhelmet results show that recombination rates in these 3 populations have diverged in fine scales but are conserved at broader scales. We also demonstrate an increase in recombination rate in areas undergoing selection despite conservation of intragenomic recombination rates at broad scales. The overlap between areas of enhanced recombination and genomic regions subject to selection likewise supports the existence of recombination modifiers which are subject to selection during rapid evolutionary change.

## Materials and methods

### Fly populations

The *D. melanogaster* populations used in this study were generated previously as part of an experimental evolution study in which wild-caught females from British Columbia were used to establish a large set of isofemale lines. From this large breeding population, 3 sets of 5 replicate populations were generated and each set was subjected over an approximately 3-year period to one of 3 experimental temperature regimes: Warm (25**°**C), Cold (16**°**C), and Temp (migration between the Warm and Cold regimes every 4 weeks; Yeaman *et al.* 2010). At the end of this period, a set of isofemale lines were established from each population replicate and maintained at a constant temperature of 20.5**°**C during a 27-month period to allow for the establishment of isogenic lines. The establishment of isogenic lines also isogenizes the genome within each line minimizing further evolutionary processes (Cooper *et al.* 2012). These resulting populations were previously shown to exhibit heritable differences in recombination rate, as measured using a set of visible markers on chromosome 3R, with those from the Warm and Cold regimes exhibiting the highest and lowest rates, respectively, and the Temp regimes exhibiting an intermediate rate (Kohl and Singh 2018).

### Crosses and variant calling

Generation, sequencing, and variant calling of haploid embryos were as described previously (Winbush and Singh 2021). Females from isogenic lines representing the 3 sets of 5 replicate populations for each temperature regime were crossed to males bearing the male-sterile *ms(3)K81* mutation. The resulting progeny from this cross are haploid with all genetic material being maternally derived (Fuyama 1984; Langley *et al.* 2011). Haploid embryos surviving to the first instar larval stage (∼1% of progeny) were collected from each isogenic line resulting in 48 individual embryos for each temperature regime (hereafter referred to the Cold, Warm, and Temp populations). This methodology is advantageous in that it allows for the isolation and sequencing of haploid genomes in which each sample represents a single haplotype derived from isogenic populations and therefore does not require haplotype phasing. This technique has been previously used in the analysis of genetic variation between population samples (Pool *et al.* 2012). The use of haploid embryos allowed for a single haplotype per sample with no haplotype phasing requirement*s*.

For preparation and sequencing of DNA libraries from individual embryos, embryonic DNA was extracted and amplified using the REPLI-g Midi kit (Qiagen, Valencia, CA, USA) according to the manufacturer’s instructions. DNA amplification was performed for 16** **h at 30**°**C followed by heat inactivation at 65**°**C for 3** **min. DNA fragmentation was performed using NEBNext dsDNA Fragmentase (New England Biolabs, Ipswich, MA, USA). PCR enrichment steps used NEXTFLEX DNA barcoded adapters and primers (Applied Genomics, Waltham, MA, USA) coupled with Phusion HS FLEX DNA polymerase (New England Biolabs, Ispwich, MA, USA).

Barcoded DNA libraries were sequenced on 3 lanes using the Illumina HiSeq2000 (48 samples per lane) producing 100**-**bp paired-end reads. On average, 1** µ**g of each sample was loaded on their respective lanes and run with standard Illumina protocols.

The resulting reads were mapped to the *D. melanogaster* reference genome (Flybase, r6.22) using the MosaikAligner toolset (v2.2.3; Lee *et al*. 2014). After removing libraries with poor alignment, a total of 137 libraries remained for downstream variant calling. SNP calling was performed using both Freebayes (v.1.2.0) and Joint Genotyper for Inbred Lines (JGIL; v.1.6) software (Marth *et al.* 1999; Garrison and Marth 2012; Stone 2012). SNPs called by both methods were filtered based on coverage, quality scores (phred-encoded score** **>** **15), and allele consistency resulting in 835,207 SNPs in our dataset.

### Generation of recombination estimate maps

Recombination rate estimates for the 3 populations were performed using the LDhelmet (v.1.10) program. Like LDhat but tailored to *Drosophila*, LDhelmet utilizes a reversible jump Markov Chain Monte Carlo (rjMCMC) model to estimate the population-scaled recombination rate, **ρ**, based on patterns of LD among SNPs within a set of haplotypes comprising a population. Values of **ρ** (4N_e_r; N_e_ =** **effective population size; r** **=** **recombination rate) are partially based on those required to produce observed rates of LD decay between loci within a population (Li and Stephens 2003; McVean *et al.* 2004; Auton and McVean 2007; Chan *et al.* 2012). In addition, LDhelmet is suited to handling larger sample sizes, missing alleles, and the higher SNP densities common in *Drosophila* (Chan *et al.* 2012).

To generate FASTA files for input into LDhelmet, we used a combination of shell tools and bcftools (v.1.9-67-g626e46b; Li 2011) to separate our initial VCF file by population (Cold, Warm, and Temp) and chromosome arm. For each population, a FASTA consensus sequence containing the corresponding SNPs at the appropriate locations was created for each sample with invariant sites containing the reference sequence (Flybase, r6.22). Sites for which samples lacked coverage were masked with N’s to denote missing data. This was performed separately for all 5 long chromosome arms resulting in a set of haplotypes for each population and chromosome arm. As a result, complete population-specific FASTA files for each chromosome contained 42, 47, and 48 individual sample haplotypes for the respective Cold, Warm, and Temp populations.

We ran LDhelmet individually for each chromosome arm and population using the default parameters for the find_conf and pade modules. Theta values were estimated using the PopGenome package implemented in R (v.2.7.5; Pfeifer *et al.* 2014) with values at 0.001 being observed for the 3 populations. For the mutation transition matrix (matrix specifying transition probabilities from one allele to another), we used the values previously derived from the Raleigh population in Chan *et al.* (2012) as we expect our populations to have similar values based on the similar outgroup reference genomes. We ran the rjmcmc module for 1,000,000 iterations with a 100,000-iteration burn-in. The choice of block penalty has a large impact on recombination estimate map smoothing. The previous studies in *Drosophila* species utilized a block penalty of 50 and found no effect of different block penalties on overall results (Chan *et al.* 2012; Smukowski *et al.* 2015). We therefore used a block penalty of 50.

### Comparison to empirical recombination maps

We utilized empirical recombination maps that were previously generated for *D. melanogaster* through large-scale crosses and sequencing of a set of inbred strains (Comeron *et al.* 2012). Recombination maps from these data were generated at 100- and 20-kb windows. Comparison to our LD-based data at similar intervals was performed by first converting our population-scaled rates (**ρ**/bp) to cM/Mb using methods previously described (Chan *et al.* 2012; Smukowski *et al.* 2015) and averaging over the same window sizes. Our converted maps were then compared to the empirical maps using Spearman’s rank coefficient and are reported as Spearman’s rho and *P*-value for the indicated window size.

### Comparison of recombination rates between populations and to genomic features

Comparisons of recombination rates to genomic features and between population pairs for each chromosome arm were carried out using wavelet analysis. An advantage of wavelet analysis is that it allows correlations between features to be compared at different scales independently (Spencer *et al.* 2006; Chan *et al.* 2012). A detailed description of this methodology is available in Spencer *et al.* (2006) and is only briefly described here: Essentially, a wavelet-transform extracts frequency components from a series of observations by transforming the waveform into a series of detail and smooth coefficients. These coefficients both represent signal variation and a smooth approximation of the original signal, respectively, at increasing scales. With this methodology, our recombination maps represent the different series of observations which are treated as discrete “time series” data sets. The analysis is performed at increasingly broader scales on the maximum available number of observations for each chromosome that represents 2^*n*^ observations and *n* scales. Collectively, these methods allow the comparison of recombination and genomic features at fine and broad scales, eliminating the need to choose arbitrary window sizes. Furthermore, the signal variation at different scales remains independent since detail coefficients at different scales are orthogonal to each other (Spencer *et al.* 2006). This allows for correlation analysis on detail coefficients, and comparison of results at different scales using linear models. For this study, we limited our analysis to the discrete wavelet transform and resulting detail coefficients after using the Haar wavelet, utilizing the methods and scripts in Spencer *et al.* (2006; also performed in Chan *et al.* 2012). In our analysis, the recombination rates and genomic features including gene content (calculated as the proportion of nucleotides in each window that encode exons) and nucleotide diversity were binned as means in 25**-**kb windows and log transformed. We also included read depth (binned in 25**-**kb windows) to control for the effects of differences in sequencing depth between samples on nucleotide diversity (Spencer *et al.* 2006). Data were analyzed for each of the 5 chromosome arms for the 3 populations. We also carried out a comparison of recombination maps between population pairs (Cold vs Warm, Cold vs Temp, and Warm vs Temp).

In addition, we compared average recombination rates among populations across all windows at conventional scales (50, 100, 200, and 500** **kb) for each chromosome arm using ANOVA. Pairwise comparisons between populations for statistically significant results were carried out using Tukey’s HSD test.

## Results

### Comparison to empirical recombination rates

We utilized the LDhelmet software to infer recombination rate histories within our temperature-derived experimental evolution populations (Cold, Warm, and Temp) of which the resulting fine-scale recombination maps are shown in Supplementary Fig. 1. In addition to recombination rate, these populations were previously shown to have diverged in several phenotypes including cell membrane lipid composition, cell size, metabolism, fecundity, developmental plasticity, and thermal tolerance (Cooper *et al.* 2012; Condon *et al.* 2014, 2015; Adrian *et al.* 2016; Le Vinh Thuy *et al.* 2016; Alton *et al.* 2017; Kohl and Singh 2018). This, coupled with observed divergence in SNP allele frequencies between the populations, allows for an excellent opportunity to utilize LD-based methodologies to infer historical recombination rates within the different populations. We also note that these estimates of recombination will be based on the shared evolutionary history of these populations, given they were founded from the same source, in addition to changes in recombination during the course of the experimental evolution study. LD-based methods of assessing recombination rate offer practical advantages over empirical methods such as controlled crosses and extensive genotyping which are often laborious. However, as noted in Smukowski *et al.* (2015), statistical LD-based methods have their limitations due to other factors that influence LD, including sudden demographic changes, genetic drift, changing mutation rates, and selection (Smith and Fearnhead 2005; Slatkin 2008; Dapper and Payseur 2018; van Eeden *et al.* 2021). Furthermore, LD-based methods are typically utilized to infer historical recombination rates over long timespans into the past (Hermann *et al.* 2019; van Eeden *et al.* 2021) which contrasts the relatively short experimental-evolution time frame of our populations.

We therefore compared our LDhelmet data for the 3 populations to recombination maps produced based on publicly available empirical data (Fiston-Lavier *et al.* 2010; Comeron *et al.* 2012). Given that populations used to derive the empirical datasets were not subject to an experimental-evolution scheme, it is inappropriate to make direct inferences on how well LDhelmet can approximate empirical results based on results obtained from our experimental datasets. However, the quality of the LDhelmet-generated recombination maps from our experimental datasets can be compared to the empirical maps with the expectation that features common to all *D. melanogaster* recombination maps are present. Comparisons based on average recombination rates at 100- and 200**-**kb windows showed significant correlation at both scales (Table 1). We note stronger correlation values (Spearman’s rho) at 200** **kb for all 3 populations and all 5 major chromosome arms (Table 1b). In all 3 populations, the highest correlations were found overall on chromosome arms 2L and 3L while chromosome X had the weakest correlations. In comparison to results obtained by the Chan *et al.* (2012) study which utilized a different empirical dataset obtained from flybase, and populations derived from Raleigh, United States (RA) and Gikongoro, Rwanda (RG), we note slightly weaker correlations overall of our 3 populations to the empirical dataset we utilized. For example, mean correlations at 200** **kb obtained by Chan *et al.* for RA and RG were 0.74, 0.77, 0.64, 0.69, and 0.67 for chromosomes 2L, 2R, 3L, 3R, and X, respectively, while mean values for the Cold, Warm, and Temp populations were 0.71, 0.50, 0.72, 0.61, and 0.43 for the same respective chromosomes. The use of different empirical datasets and different populations accounts for some of these differences; however, we should note that our experimental evolution design, which made use of different rearing temperatures, likely affects recombination rates and is therefore likely also responsible.

**Table 1.** Comparison of LDhelmet-estimated recombination rates and empirical data derived from Comeron *et al.* (2012) for the 3 populations (Cold, Warm, and Temp) for the 5 major chromosomes at the (a) 100**-**kb interval and (b) 200**-**kb interval.

	Cold	Warm	Temp
(a)			
2L	0.61, *P* < 0.0001, 613	0.66, *P* < 0.0001, 675	0.63, *P* < 0.0001, 724
2R	0.50, *P* < 0.0001, 400	0.48, *P* < 0.0001, 408	0.48, *P* < 0.0001, 450
3 L	0.57, *P* < 0.0001, 559	0.61, *P* < 0.0001, 581	0.65, *P* < 0.0001, 623
3R	0.55, *P* < 0.0001, 455	0.54, *P* < 0.0001, 468	0.53, *P* < 0.0001, 503
X	0.32, *P* < 0.0001, 385	0.38, *P* < 0.0001, 402	0.42, *P* < 0.0001, 430
(b)			
2L	0.72, *P* < 0.0001, 1,227	0.72, *P* < 0.0001, 1,351	0.70, *P* < 0.0001, 1,449
2R	0.52, *P* < 0.0001, 800	0.47, *P* < 0.0001, 816	0.52, *P* < 0.0001, 901
3 L	0.70, *P* < 0.0001, 1,122	0.72, *P* < 0.0001, 1,167	0.75, *P* < 0.0001, 1,251
3R	0.62, *P* < 0.0001, 910	0.61, *P* < 0.0001, 937	0.60, *P* < 0.0001, 1,007
X	0.38, *P* < 0.0001, 775	0.44, *P* < 0.0001, 809	0.46, *P* < 0.0001, 864

Entries (separated by commas) as follows: Spearman’s rho, adjusted *P*-value based on Bonferroni’s correction, and average number of SNPs per interval.

We note several features across our datasets that are similar to those found in the empirical dataset at the 100**-**kb window size. For example, we observed decreased recombination rates toward centromeric regions in our 3 populations as has been noted in other studies (Comeron *et al.* 2012; reviewed in Nachman 2002) and a notable spike in recombination on chromosome 2L near the 10**-**Mb position (Chan *et al.* 2012), which we note also occurred in the Cold and Temp populations but not the Warm population (Supplementary Fig. 2). Uniform variation in recombination rate is noted on the X chromosome in all 3 populations and the large-scale fluctuations noted in the empirical dataset are less prominent. The mean recombination rates for all 200**-**kb windows on the X chromosome were also lower for the 3 populations overall (2.89, 2.88, and 2.88 cM/Mb for the Cold, Warm, and Temp populations, respectively, vs 2.95 cM/Mb for the empirical dataset). In summary, our data suggest a good estimation of recombination rates in our 3 populations with some expected variation which is further explored below.

### Recombination rate variation between populations

**Fig. 1.**
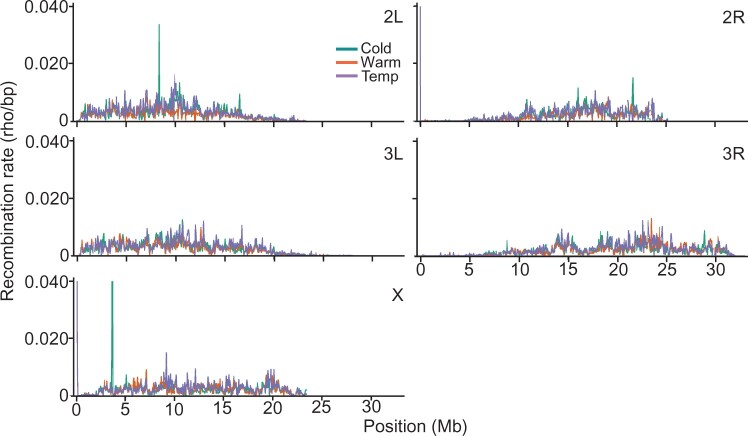
LDhelmet recombination rate estimate comparisons between the 3 populations (Cold, Warm, and Temp) for the 5 major chromosome arms averaged in 50-kb windows.

**Fig. 2.**
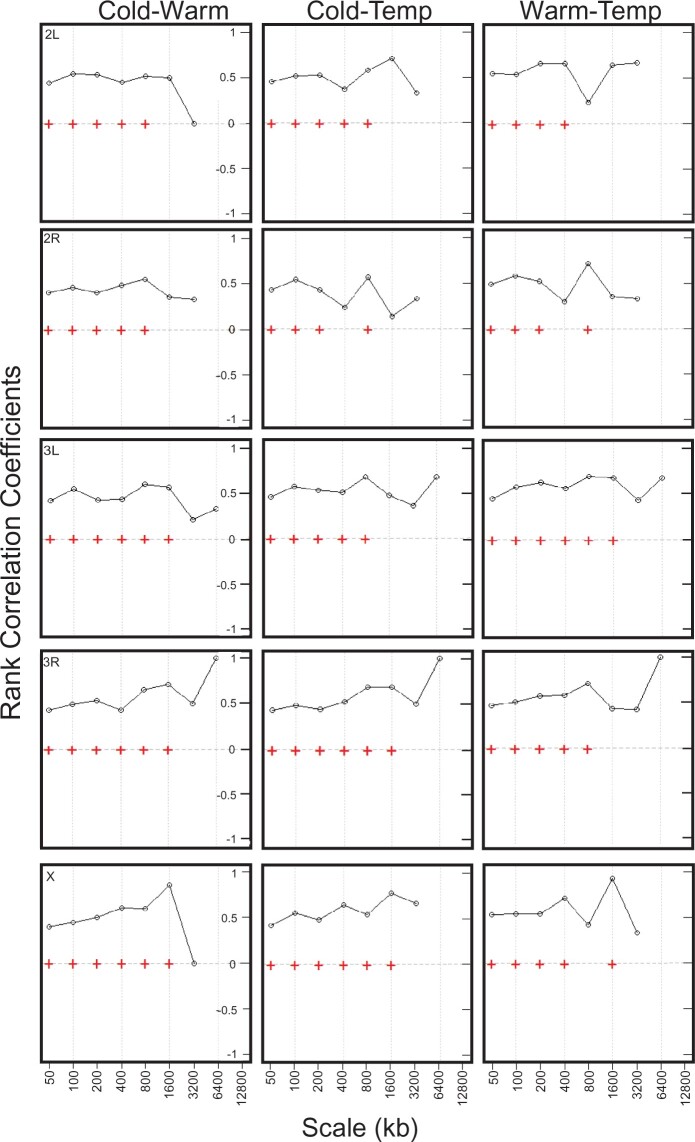
Pairwise rank correlation coefficients between detail wavelet coefficients, derived from the population-specific recombination maps, at the indicated scales for the 3 population comparisons (Cold vs Warm, Cold vs Temp, and Warm vs Temp). Each row represents a chromosome arm. Crosses denote correlations that are significant at the 1% level (Kendall’s rank correlation).

Based on the experimental evolution design that generated these populations, we expect to find variation in historical recombination rate patterns between the 3 populations. The previous studies found variation in recombination rate at fine scales between different *Drosophila* species and different *D. melanogaster* populations (Chan *et al.* 2012; Smukowski *et al.* 2015; Adrion *et al.* 2020). We therefore tested our populations for variation in recombination rate at fine and broad scales. For purposes of this study, we classify “fine scale” to include window sizes of 50** **kb and less along with any associated features such as “hot/warm spots” (detailed below), and “broad scale” to include window sizes above this level. Recombination maps at the 50**-**kb scale are shown in Fig. 1 with broader maps in Supplementary Fig. 3. Given our population comparisons (Cold vs Warm, Cold vs Temp, and Warm vs Temp) and number of chromosome arms, repeated measures, and comparisons of correlation using Spearman’s rho at different scales can produce results that are difficult to parse. We therefore carried out a wavelet analysis on our data, which allow for a more detailed analysis of frequency components at different scales (see *Materials and Methods*). We examined pairwise correlations between the detail coefficients, resulting from the Haar wavelet transform of our recombination maps. This allowed us to assess covariance of our population comparisons at the resulting scales. With this analysis, we were able to assess pairwise correlations for the population comparisons from 50**-**kb to 1.6**-**Mb windows after which statistical power is lost (Fig. 2). We note that for all population comparisons (Cold vs Warm, Cold vs Temp, and Warm vs Temp), and chromosome arms (2L, 2R, 3L, 3R, and X), recombination maps show significant correlation at most scales up to 1.6 mb with the most notable exceptions on chromosome 2R for the Cold vs Temp and Warm vs Temp comparison. However, we observed consistently higher correlations at broad scales across all comparisons for most chromosome arms. This is most notable through the 800**-**kb window size for chromosomes 3L, 3R, and X. The highest correlations were at the 1.6-mb scale (the highest available scale with statistical power for all chromosome arms) for all comparisons and the majority of chromosome arms except for chromosome 2R in the Warm vs Temp comparison and Cold vs Temp comparisons. There were also slightly higher correlations at the fine scale of 50** **kb and broad scale of 100** **kb for the Warm vs Temp comparisons for the 5 chromosome arms.

**Fig. 3.**
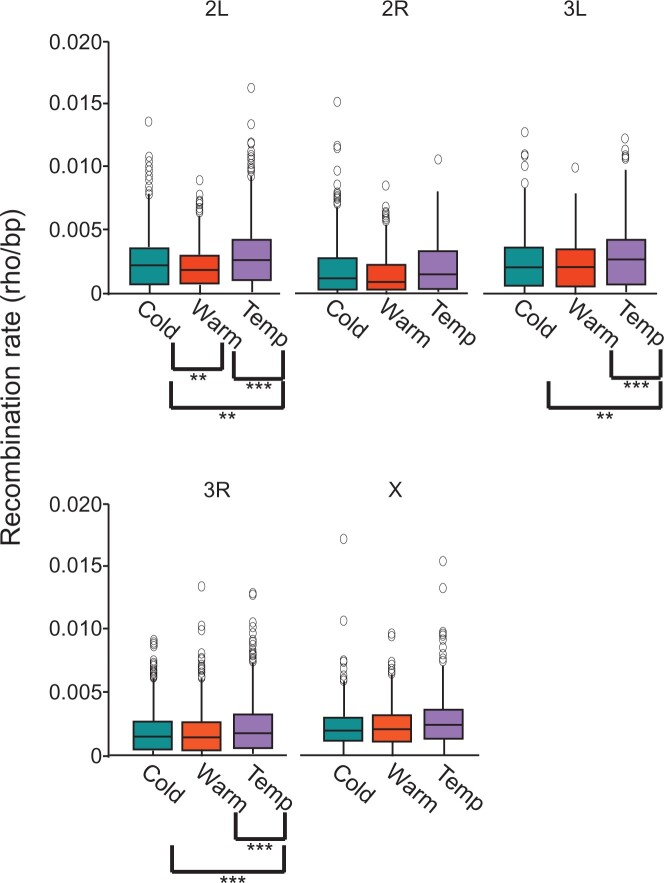
Boxplots comparing recombination rate distribution across all windows (50-kb window size) for the 5 major chromosome arms of the Cold, Warm, and Temp populations. Most extreme outliers are omitted from figure. Population comparisons with significant differences in recombination rates are indicated with brackets. **P < 0.05, ***P < 0.001 (Tukey’s HSD test for all comparisons).

Outside of wavelet analysis, we also compared mean recombination rates across all windows at conventional window sizes (50, 100, 200, and 500** **kb) between the 3 populations. We note significant differences at 50** **kb for chromosome arms 2L, 3L, and 3R (*P* <0.0001; one-way analysis of variance). Post hoc comparisons of population pairs confirm these results and would suggest higher recombination rates in the Temp population on chromosomes 2L, 3L, and 3R (Fig. 3). At increasingly broader scales, significant differences in recombination rates on chromosome arms 2L, 2R, 3L, and 3R were observed at 100** **kb (*P* <** **0.01 in all instances; one-way analysis of variance), while at 200** **kb, significant differences are observed on chromosome arms 2L, 2R, and 3R (*P* <** **0.05 in all instances; one-way analysis of variance) and at 500** **kb on chromosome 2L (*P* <** **0.05; one-way analysis of variance). In most cases, higher rates in the Temp population appear to be the main contributor to these differences based on post hoc pairwise comparisons (Supplementary Fig. 4). Collectively, these data indicate the highest historical rates in the Temp population overall.

### Nucleotide diversity and genomic feature correlates

**Fig. 4.**
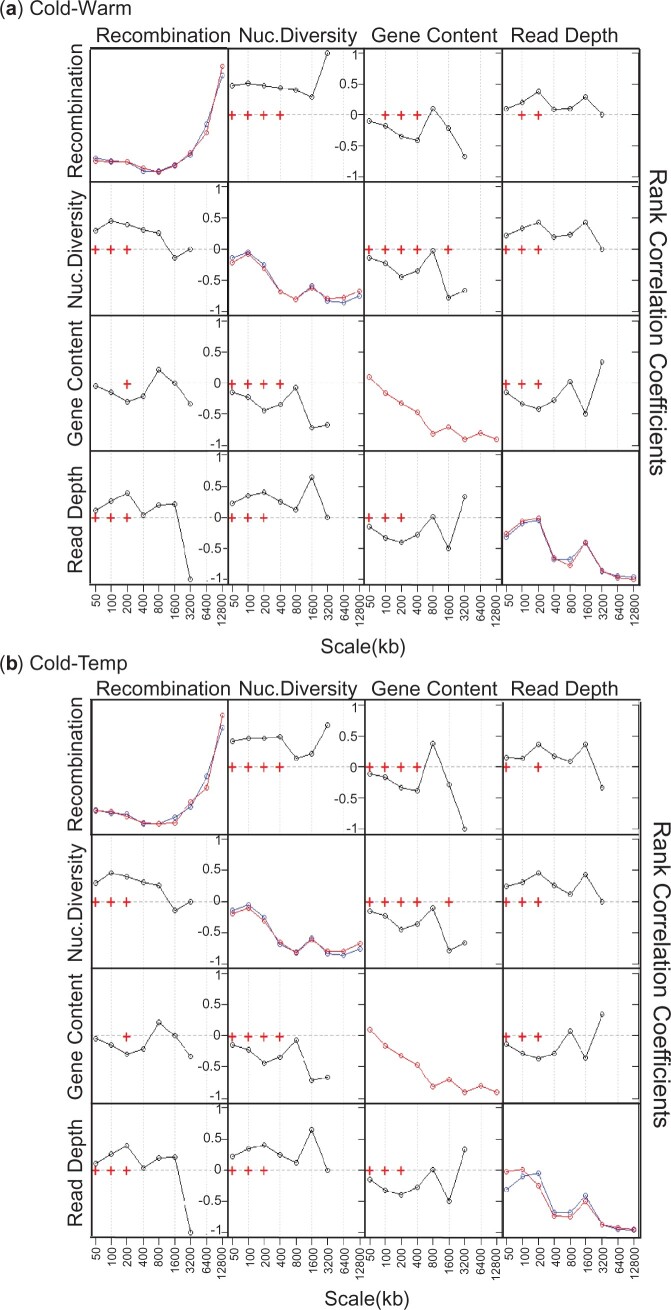
Power spectra and pairwise rank correlation coefficients between detail wavelet coefficients, derived from the population-specific recombination maps for the 3 populations (Cold, Warm, and Temp) and the indicated genomic features for chromosome 2L. Off-diagonal plots indicate rank correlation coefficients between detail wavelet coefficients, derived from the population-specific recombination maps, and the genomic features. Crosses denote correlations that are significant at the 1% level (Kendall’s rank correlation). To utilize even comparisons, the Cold population is utilized in both (a) and (b) and represents the matrix plot left and bottom of the diagonal with the Warm population (a) or Temp population (b) right and above the diagonal. Diagonal plots denote the wavelet power spectra of each indicated feature with the Cold population in blue and the Warm population (a) or Temp population (b) indicated in red.

Associations between recombination rate, nucleotide diversity, and other genomic features have previously been noted at varying levels in several species, including those of *Drosophila* (Begun and Aquadro 1992; Cai *et al.* 2009; Charlesworth and Campos 2014; Corbett-Detig *et al.* 2015; Kartje *et al.* 2020). Furthermore, decreases in nucleotide diversity have been associated with the effects of linked selection. To this end, we extended our wavelet analysis to examine pairwise correlations between detail coefficients for recombination rates, nucleotide diversity, and genomic features including gene content and read depth (see *Materials and Methods*) for the 3 populations. The off-diagonal plots in Fig. 4 represent pairwise correlations between the detail coefficients for those features of chromosome 2L (statistical significance measured using Kendall’s rank correlation). We observe positive correlations for recombination and nucleotide diversity at fine scales (50** **kb) with either a slight increase or “leveling off” across broader scales for all 3 populations. In addition, negative correlations are observed between recombination rate and gene content at broad scales up to 400** **kb. Finally, a positive correlation between read depth and either recombination rate or nucleotide diversity is noted at fine and broad scales up to 200** **kb with loss of significance beyond this scale suggesting a weak contribution of sequencing biases to nucleotide diversity. Across the remaining chromosome arms (Supplementary Figs. 5–8) similar trends are noted with a general increase in correlation between recombination rate and nucleotide diversity across broader scales up to 800** **kb and a decrease thereafter. The exception was for chromosome 3L (Supplementary Fig. 6) in which recombination rate and nucleotide diversity exhibit increases in correlation up to at least 1.6 mb. The weakest correlations were observed in the X-chromosome (Supplementary Fig. 8) with correlation values decreasing at broader scales and becoming statistically insignificant at 800** **kb in the Temp population. This would suggest that there is only a weak link between diversity and recombination overall in this chromosome. Outside of this, however, we see a trend in the 3 populations, especially the Cold and Temp in which recombination likely contributes significantly to nucleotide diversity at various scales, most notably on chromosomes 2R and 3L (Supplementary Figs. 5 and 6). Therefore, we conclude a link between fine-scale recombination rates and linked selection in the 3 populations for the 5 major chromosomes.

**Fig. 5.**
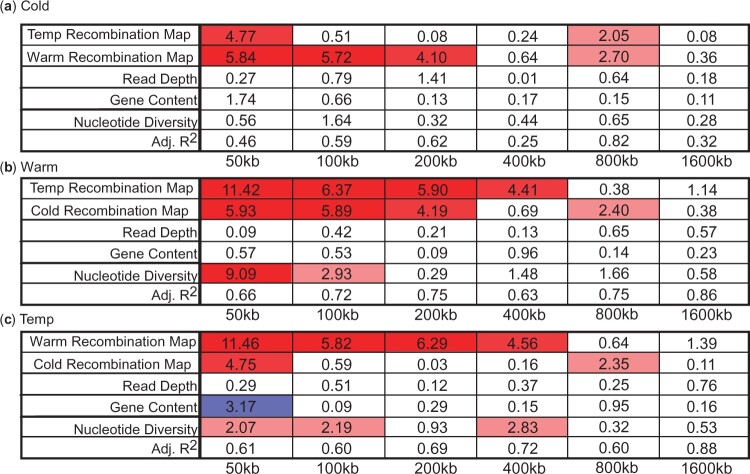
Linear model for the detail coefficients of the wavelet transforms of the population-specific recombination maps for the (a) Cold, (b) Warm, and (c) Temp populations with the detail coefficients of the wavelet transforms of the indicated features serving as covariates/predictors in chromosome 2L. Values represent the -log_10_*P*-value of the regression coefficient (*t*-test) and the adjusted *r*^2^ is included in the bottom row. Red/blue boxes indicate a significant positive/negative linear relationship between the covariate and the recombination map at that scale. Note that for each population, the other 2 remaining populations are also included as covariates/predictors.

As a final step, we performed a linear model analysis of the wavelet coefficients of the recombination maps of each population using the wavelet coefficients of the genomic features (nucleotide diversity, gene content, and read depth) and the 2 remaining populations as predictors (Spencer *et al.* 2006; Chan *et al.* 2012). For chromosome 2L in each population (Fig. 5), the largest predictors of recombination rate were the 2 remaining populations and nucleotide diversity. For example, in Fig. 5b, the most statistically significant covariates for the Warm population maps were the Cold and Temp recombination maps and nucleotide diversity, with nucleotide diversity becoming statistically insignificant at the 200**-**kb scale. Indeed, the influence of nucleotide diversity was limited to mostly to lower/fine scales in the 3 populations. In addition, the Temp recombination map was a better predictor for the Warm recombination map and vice versa suggesting those 2 recombination maps are better correlated. Likewise, the Warm and Cold recombination maps were the better predictors of each across a wider range of scales while statistically significant values between the Cold and Temp population were limited to the 50**-**kb fine scale and 800**-**kb broad scale. This result would suggest greater divergence in recombination rate for the Cold population. Examining the remaining chromosome arms, several differences were noted (Supplementary Figs. 9–12). For example, in chromosome 2R (Supplementary Fig. 9), nucleotide diversity was a larger predictor for the Temp recombination map across all scales to 400** **kb, and the Cold recombination map was again a poor predictor for the other maps. For chromosome 3L, the influence of nucleotide diversity was less pronounced while the influence of the map from the Temp population was a better predictor for both the Cold and Warm maps (Supplementary Fig. 10). These patterns persisted for chromosome 3R and X but were less prominent for the X chromosome where the Warm and Temp recombination maps were better correlated (Supplementary Figs. 10–12). Collectively, these results demonstrate nucleotide diversity and the other maps as strong predictors of recombination rates at fine and relatively broad scales.

### Identification and mapping of hotspots

Current evidence suggests that *Drosophila* both lack recombination hotspots on the same scale as observed in humans and experience generally higher levels of background recombination (Myers *et al.* 2005; Singh *et al.* 2009; Comeron *et al.* 2012; Manzano-Winkler *et al.* 2013). However, regions exhibiting large-scale heterogeneity in recombination rate, including areas of recombination elevated multifold over background, have been observed in *D. melanogaster* and *Drosophila pseudoobscura* (Cirulli *et al.* 2007; Singh *et al.* 2009; Comeron *et al.* 2012). At fine scales, the examination of regions between 0.5 and 6.8** **kb in LDhelmet-estimated data identified 10 putative hotspots in the *D. melanogaster* RA and RG populations and 19 putative hotspots in *D. pseudoobscura* (Chan *et al.* 2012; Smukowski *et al.* 2015). We thus wanted to investigate whether we could find similar evidence of putative “warm spots” in our 3 populations. Searching for regions between 0.5 and 7** **kb in which recombination rate exceeded 10 times the background average for that chromosome arm resulted in the identification of 9, 11, and 13 putative warm spots in the respective Cold, Warm, and Temp populations (Table 2a–c). These warm spots included both genic and intergenic regions with no overlap observed between the 3 populations. It should be emphasized that these warm spots are distinct from mammalian hotspots which are highly punctate, stable, number in the thousands, and are typically flanked by long haplotype blocks. In addition, mammalian hotspots have been verified at the molecular level to physical cross-over events using molecular techniques such as sperm typing (Paigen and Petkov 2010). Our warm spots, in contrast, have not been verified with either molecular techniques or additional statistical software (e.g. Wall and Stevison 2016). Nevertheless, the low number, less punctate, and less stable nature of our identified warm spots is consistent with other studies examining this issue in *Drosophila* species (Manzano-Winkler *et al.* 2013). Even so, these results should be considered preliminary and provisional.

**Table 2.** Putative warm spots identified in the 3 populations (a) Cold, (b) Warm, and (c) Temp based derived from LDhelmet recombination rate estimates.

Chr	Start	End	Length (bp)	Genes/features	Divergent comparisons
(a) Cold					
2L	12637749	12638263	514	Ref2*	C vs T, W vs T
2L	14821519	14822266	747	Intergenic	C vs T
2R	16113560	16115635	2,075	Asph*	C vs W, W vs T
3R	25531288	25533084	1,796	Msi*	C vs W, C vs T
3R	21649086	21650209	1,123	E2f1*	C vs W, C vs T, W vs T
3R	8839809	8841479	1,670	pyd*	C vs T, W vs T
X	3652923	3655159	2,236	AstA-R1*	C vs T, W vs T
X	14851589	14858386	6,797	CG9521, Flo2*, CG9519, CG32593, CR46391	C vs T
X	22766036	22766595	559	CR44997*	C vs W, C vs T, W vs T
(b) Warm					
2R	19119344	19119925	581	5-HT1A*	None
2R	25256320	25262697	6,377	Intergenic	C vs W, W vs T
2R	21485446	21486594	1,148	Sara*	C vs T, W vs T
3L	1900669	1901939	1,270	Hip1*	None
3L	12770565	12771232	667	dsb*	None
3L	4534477	4537466	2,989	CG11353*	C vs W
3R	18001031	18001641	610	Ugt303B3*, Intergenic	C vs W
3R	23533398	23534851	1,453	cpo*	W vs T
X	122027	123171	1,144	*CR40469*, Intergenic	C vs W, W vs T
X	7111274	7114094	2,820	Intergenic	C vs W, W vs T
X	9102582	9104257	1,675	CG7766*, Bx42*, Intergenic	W vs T
(c) Temp					
2L	11974677	11975569	892	Intergenic	None
2L	10013333	10015593	2,260	*CG13131*	None
2L	** **14221991	14223009	1,018	Dyrk2*	None
2L	8806997	8807987	990	Intergenic	None
2R	25266512	25267975	1,463	Intergenic	C vs W, W vs T
2R	25267975	25274221	6,246	Intergenic	C vs W, W vs T
2R	18628050	18629052	1,002	*Eip55E*	None
3L	11366585	11367305	720	Intergenic	W vs T
3R	23163052	23163810	758	Intergenic	C vs T, W vs T
X	9185553	9192553	7,000	Mei-P26*, Intergenic*, CG12115, CG12057*	C vs W, W vs T
X	12107507	12109867	2,360	CR43960*	C vs T, W vs T
X	17812622	17813175	553	OdsH*	C vs T
X	20118472	20119565	1,093	Intergenic	C vs T

Columns depict the chromosome, start, end, base-pair length, and associated features of each warm spot. Asterisks denote genes previously identified as divergent. Final column depicts population comparisons for which the regions encompassing the warm spot in question were previously identified as divergent between the populations. C vs W: Cold vs Warm; C vs T: Cold vs Temp; W vs T: Warm vs Temp.

### Increased recombination rate in regions undergoing selection

We previously identified multiple chromosomal regions that were divergent in allele frequencies in the 3 population comparisons (Cold vs Warm, Cold vs Temp, and Warm vs Temp) using a 100**-**kb sliding window approach that took linkage into account (Winbush and Singh 2021). As these represent regions that were likely subject to selection during the experimental evolution period, we expected the warm spots we identified to overlap these regions. Consistent with this expectation, most warm spots we identified did overlap with regions identified as divergent within at least one population comparison (Table 2a–c). This was most prominent in warm spots from the Cold population; however, the Warm and Temp populations also exhibited warm spots meeting this criterion. This would suggest that during our experimental evolution period, certain regions subject to selection also showed enhanced recombination. It remains to be seen whether genes in these specific regions act as recombination rate modifiers. It is notable, however, that many of the genes associated with our warm spots were previously identified as potential candidates based on differences in associated SNP allele frequencies (Winbush and Singh 2021; Table 2a–c, asterisks).

## Discussion

### Variation in recombination rate between our 3 populations

This study examined whether populations that have undergone selection to different environments as part of an experimental evolution protocol also exhibited differences in recombination rate during the experimental evolution period. By using LD-based methods to assess these historical recombination rates coupled with our experimental evolution assay, our study has demonstrated how recombination landscapes, at fine and broad scales, can change in response to selection. We assessed this in 3 *D. melanogaster* populations that had previously been subjected to 3 temperature regimes (Cold, Warm, and Temp) as part of an experimental evolution experiment and were now divergent in several phenotypes. Evidence, based on our results, suggests that the 3 populations were divergent in recombination rate at fine scales (<50 kb) during the experimental evolution period, while broad-scale recombination rates were largely conserved. In support of this, at the fine 50**-**kb scale, for each population comparison (Cold vs Warm, Cold vs Temp, and Warm vs Temp), lower correlation values were observed compared to those of broader scales (Fig. 2). However, this effect is not as apparent on chromosome 2R. Although this could indicate conservation of recombination rate at all scales on that chromosome, this spurious result could also be an effect of wavelet analysis due to limitations in the number of observations that could be included in the wavelet transform. Additional evidence showing recombination rate divergence between the 3 populations at fine scales included the slightly higher correlation values that were observed in pairwise comparisons involving the Warm and Temp populations. This result was also noticeable in our linear models in which the Temp population was a stronger covariate for the Warm population and vice versa. In addition, our post hoc analysis of average recombination rates across all windows demonstrated statistically higher recombination rates in the Temp population at fine scale (50** **kb; Fig. 3) vs broader scales (Supplementary Fig. 4). From these data, we conclude that at fine scales, the Temp population exhibits higher average recombination rates and is more closely correlated to the Warm population, which in turn, indicates greater divergence in recombination rate in the Cold population.

Interestingly, the increased recombination rates observed for the Temp population are consistent with the hypothesized changes in recombination rate for this population resulting from the fluctuating temperature regime (Kohl and Singh 2018). In fluctuating environments, allelic combinations may be beneficial in one environment and deleterious in the future environment. If this occurs in a cyclical environment with sufficiently long periodicity, and recombination rate is condition-dependent, it was thought that higher recombination rates would evolve in populations subjected to the fluctuating environment (Kohl and Singh 2018). Although this was not borne out when examining recombination rates within the 20.4 cM region of chromosome 3R between the *ebony* and *rough* markers (Kohl and Singh 2018), we do observe this in our fine-scale data. Overall, our data support the observation that the fine-scale recombination landscapes have diverged in the 3 populations in response to selection in contrast to broad-scale recombination rates.

### Limitations of LD-based methods on our datasets

The use of statistical-based LD methods is advantageous over empirical methods in terms of time and cost; however, it must be noted that these methods represent indirect estimates of recombination. Therefore, our results are highly provisional. Indeed, as noted earlier and in previous studies, patterns of LD are also influenced by other factors such as genetic drift, demographic history and/or bottlenecks, and selection. In our 3 experimental populations, genetic drift is less likely to be a factor due to the level at which we assessed fine-scale recombination rate using a pooled population approach. However, the effects of both selection and demography are potential factors.

In the case of selection, we note that LD-based methods including LDhelmet are still robust to the effects of selection with, at worst, a tendency for such methods to slightly underestimate recombination rates in these scenarios (Smith and Fearnhead 2005; Chan *et al.* 2012). However, we cannot discount the fact that differences in SNP-allele frequencies between our 3 populations (stemming from the effects of selection) might be responsible for some of the observed recombination rate variation. Addressing this issue by removal of SNPs with significant differences in allele frequencies between populations prior to implementing the LDhelmet procedure did not impact the overall results (unpublished observations), and may adversely affect the detection of the relatively recent recombination events associated with the experimental evolution period.

Concerning demography, we note that our experimental evolution experiment spans a comparatively short time course of only 3 years compared to other studies utilizing LDhelmet that compare and infer historical recombination rate differences at the species level. Also, there are no sequencing data from the progenitor population that was used to initiate our 3 populations from which comparisons can be made. However, given that LDhelmet is still robust in the context of recent selective sweeps, we remain confident that our data represent accurate estimations of the historical recombination rates during the experimental evolution period.

### Fine-scale recombination rates and selection

An overarching theme in this and other studies concerns the existence of recombination rate modifiers in *Drosophila* which become favored in response to evolutionary change. Temperature and stress represent strong selective pressures, and both have been shown to increase recombination rate in *Drosophila* (Plough 1917; Parsons 1988; Bomblies *et al.* 2015). Indirect selection for increased recombination in response to selection due to these pressures may be favored in conditions of weak epistasis (Otto and Lenormand 2002). We investigated this by examining correlations between recombination and nucleotide diversity and overlap of warm spots with regions divergent in allele frequencies between populations. Concerning the latter, we note an overlap between many of our putative warm spots and regions previously shown to be divergent in SNP-allele frequencies in the 3 pairwise population comparisons. This would suggest enrichment of recombination in areas subject to selection in response to the temperature regime. This trend is apparent in all 3 populations despite variation in the location of warm spots between the 3 populations.

With regards to nucleotide diversity, we note that recombination rate and nucleotide diversity are correlated at fine and broad scales with generally higher correlations for broader scales (Fig. 4; Supplementary Figs. 5–8). However, in our linear models, nucleotide diversity was a weaker covariate, especially at broad scales (Fig. 5; Supplementary Figs. 9–12). The correlation between recombination rates and nucleotide diversity supports the premise that low recombination rate is favored in areas undergoing background selection for polymorphisms, with the effects of linked selection contributing to reductions in nucleotide diversity in those areas.

In conclusion, though provisional, our data provide additional evidence addressing the overarching question of how fine-scale recombination landscape are affected in response to artificial selection and supports the existence of recombination-modifying genes.

## Supplementary Material

jkac208_Supplementary_Data

## Data Availability

Population-specific raw recombination tables are available in GitHub at https://github.com/ariw237/temperature_recombination Supplemental material is available at *G3* online.
