# Supplementary material for: Variation in fine-scale recombination rate in temperature-evolved Drosophila melanogaster populations in response to selection
Source: G3 (Bethesda). 2022 Aug 12;12(10):jkac208. doi: 10.1093/g3journal/jkac208 (PMC9526048; doi:10.1093/g3journal/jkac208)
Supplement: jkac208_Supplementary_Data [file jkac208_supplementary_data.docx]

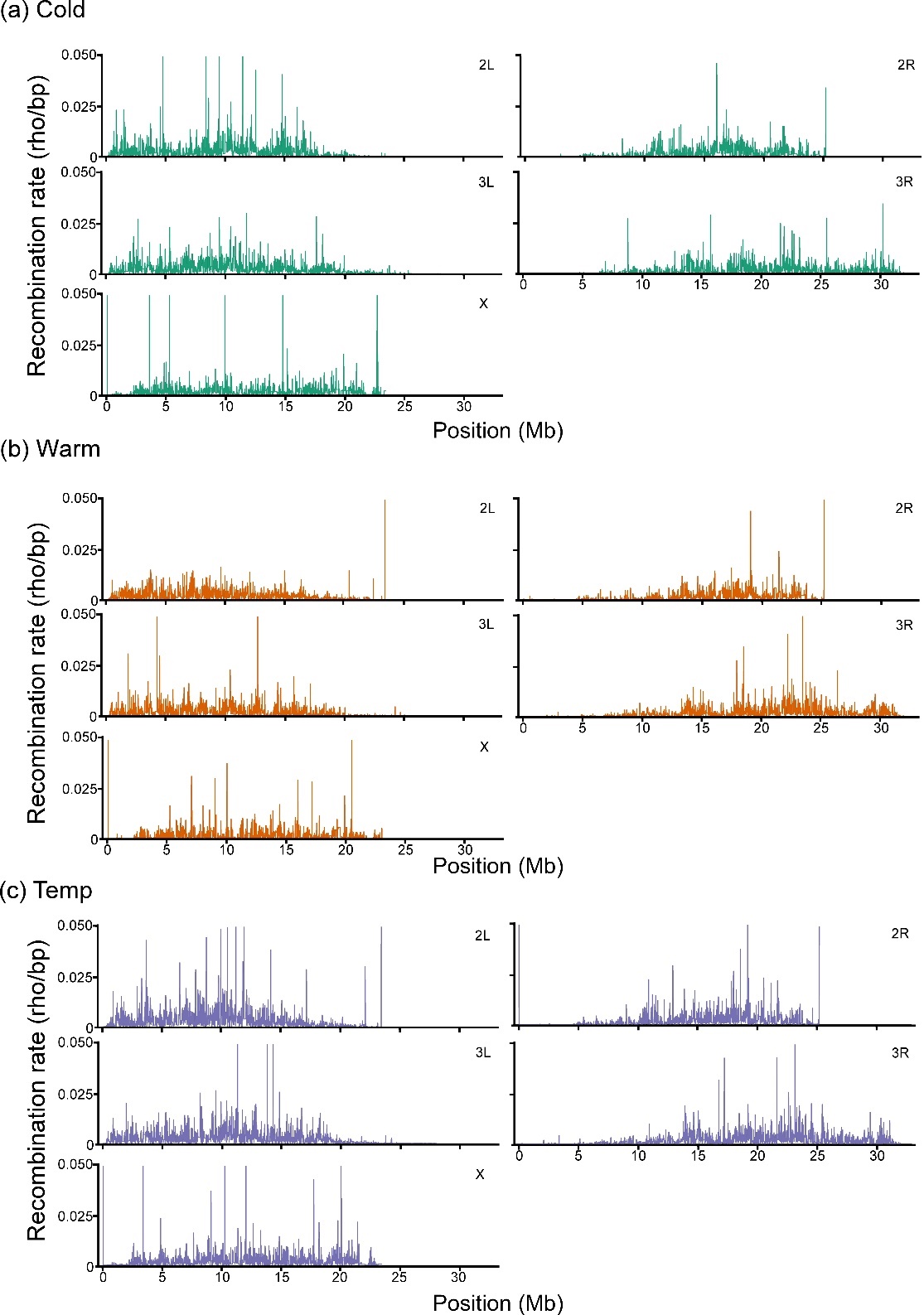


Supplementary Figure S1. LDhelmet’s fine scale recombination estimate maps. (A) Cold population. (B) Warm population. (C) Temp population.


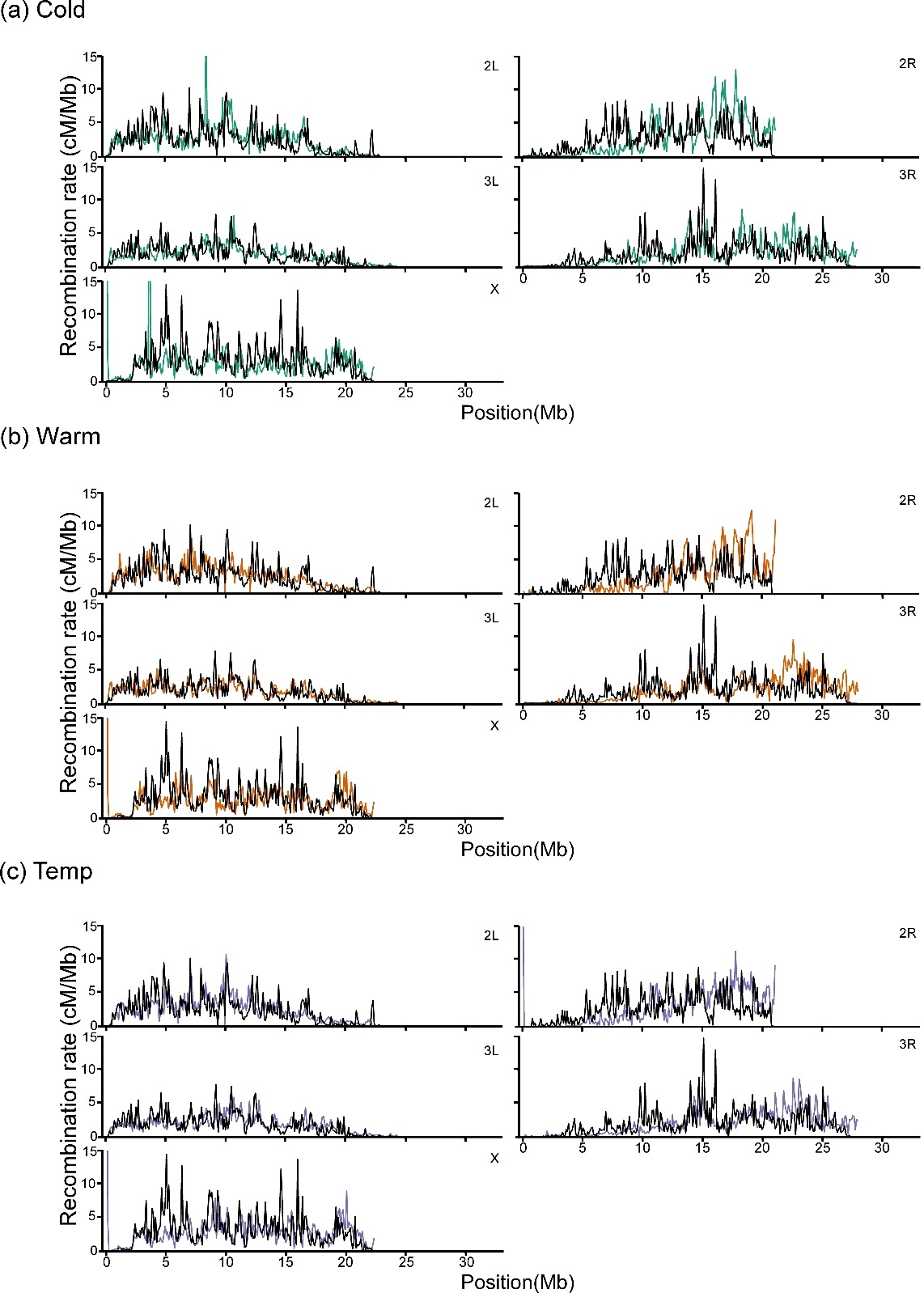


Supplementary Figure S2. A comparison of LDhelmet estimates of recombination rate to those derived from empirical methods along the five major chromosome arms. Empirical data (black lines) are derived from Comeron et al., (2012) at 100kb scales with LDhelmet estimates at the same scale. (A) Cold population LDhelmet and empirical data. (B) Warm population LDhelmet and empirical data. (C) Temp population LDhelmet and empirical data.


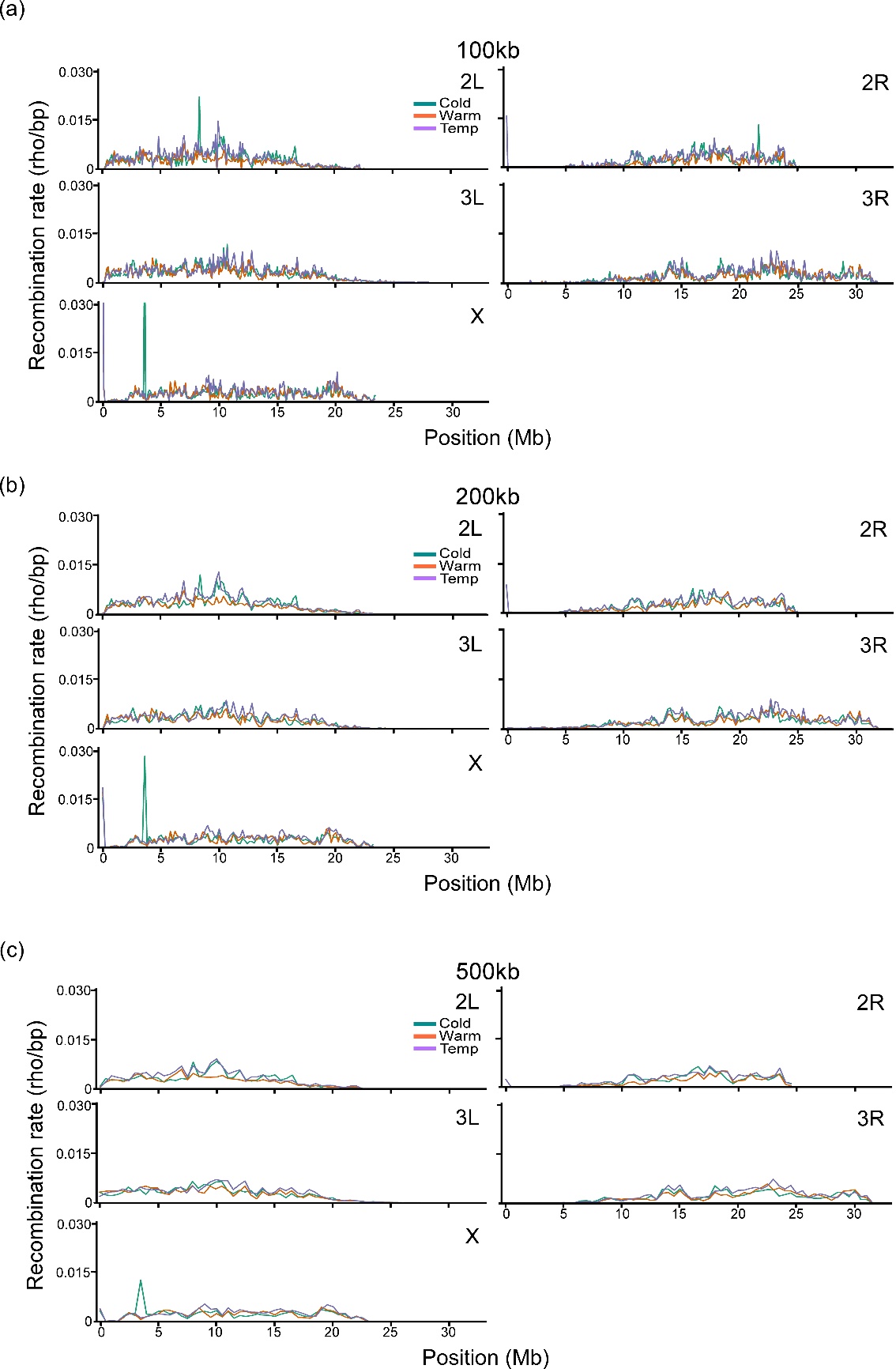


Supplementary Figure S3. LDhelmet recombination rate estimate comparisons between the three populations (Cold, Warm, and Temp) for the five major chromosome arms averaged at broader scales of 100kb, 200kb and 500kb windows.


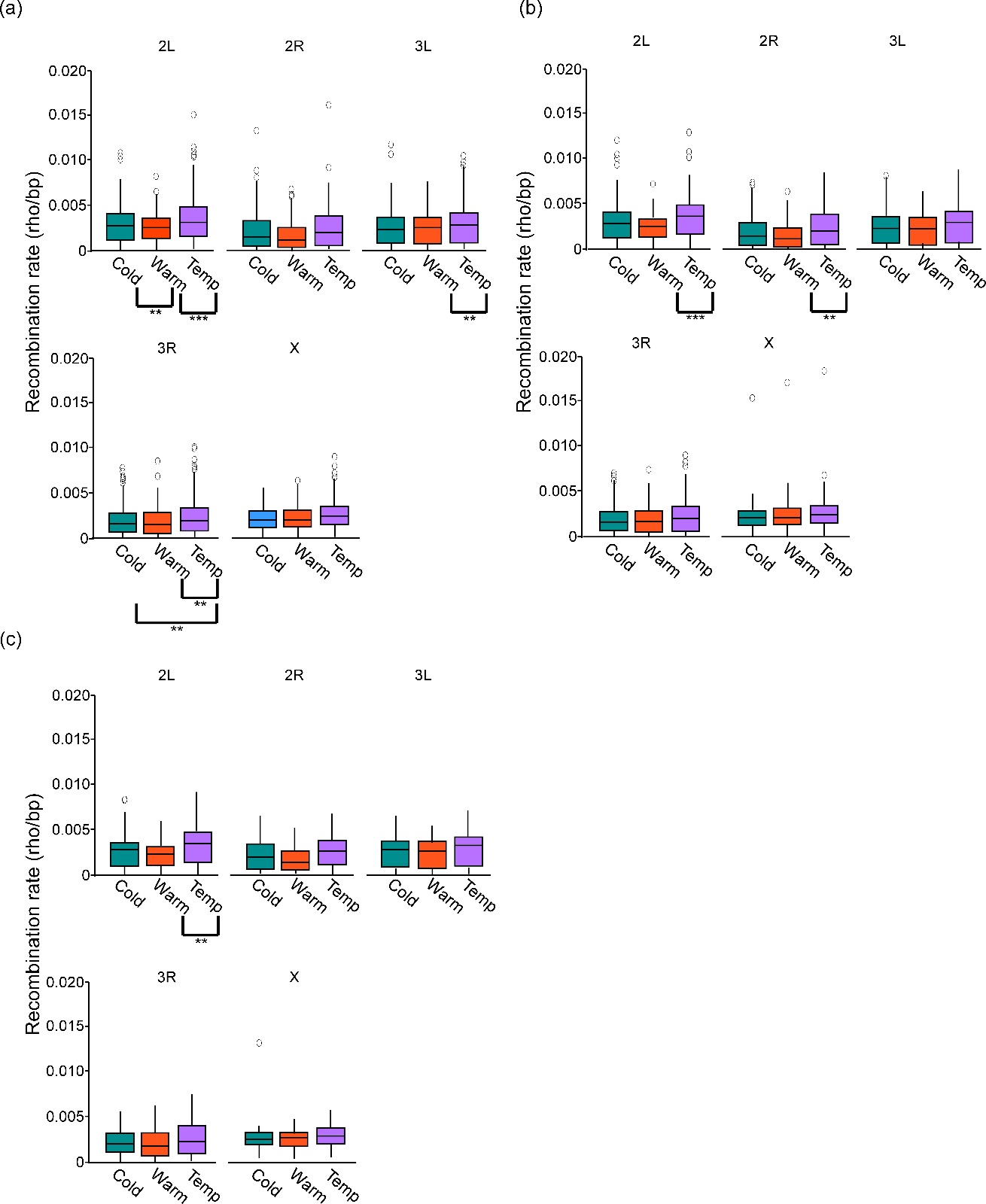


Supplementary Figure S4. Boxplots comparing recombination rate distribution across all windows for the five major chromosome arms of the Cold, Warm and Temp populations for window sizes of (a) 100kb, (b) 200kb, and (c) 500kb. Most extreme outliers are omitted from figure. Population comparisons with significant differences in recombination rates are indicated with brackets. **P<0.05, ***P<0.001 (Tukey’s HSD test for all comparisons).


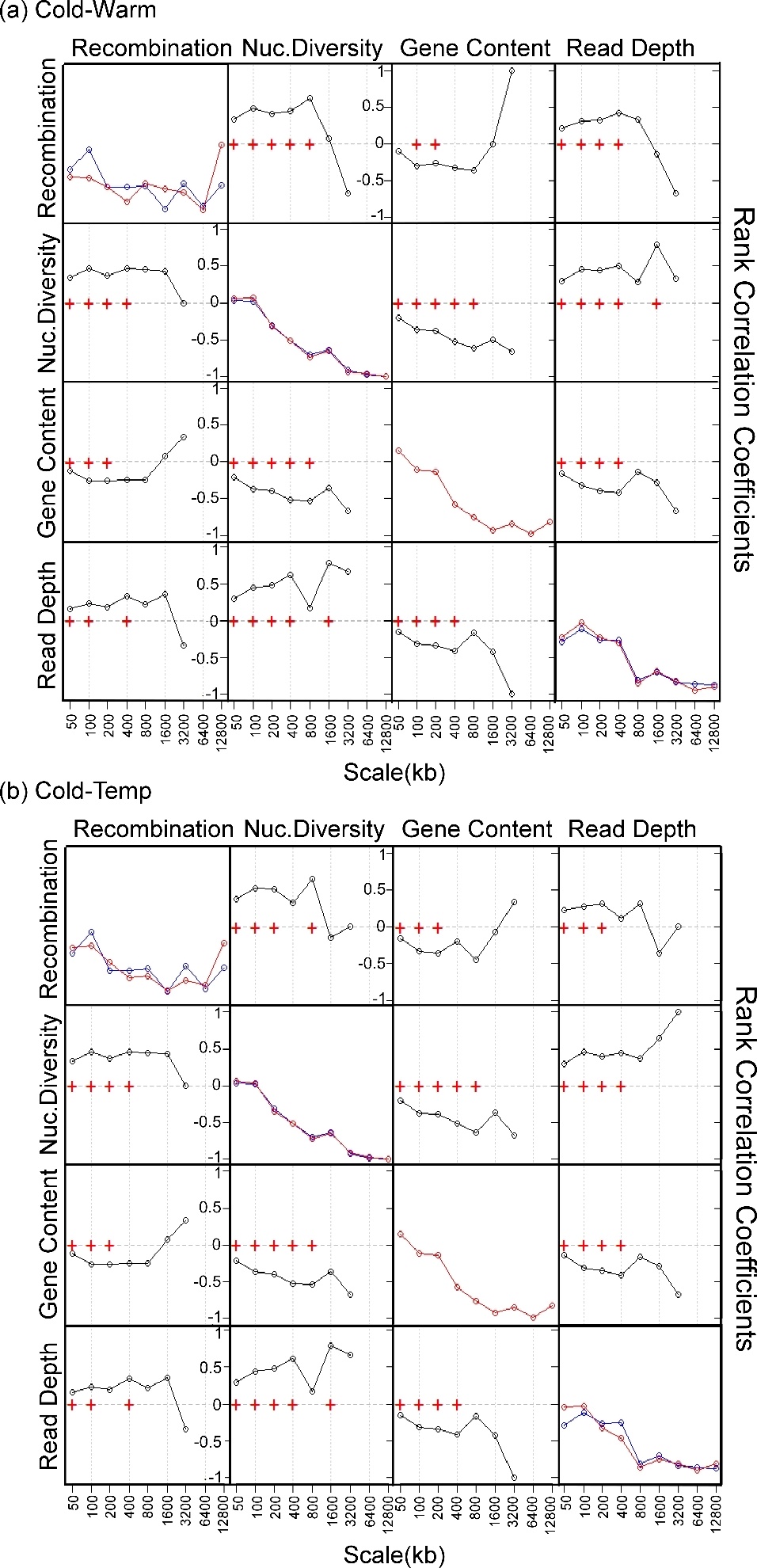


Supplementary Figure S5. Power spectra and pairwise rank correlation coefficients between detail wavelet coefficients, derived from the recombination maps for the three populations (Cold, Warm, and Temp), and the indicated genomic features for chromosome 2R. Off-diagonal plots indicate rank correlation coefficients between detail wavelet coefficients, derived from the population-specific recombination maps, and the genomic features. Red crosses denote correlations that are significant at the one percent level (Kendall’s rank correlation). To utilize even comparisons, the Cold population is utilized in both (a) and (b) and represents the matrix plot left and bottom of the diagonal with the Warm population (a) or Temp population (b) right and above the diagonal. Diagonal plots denote the wavelet power spectra of each indicated feature with the Cold population in blue and the Warm population (a) or Temp population (b) indicated in red.


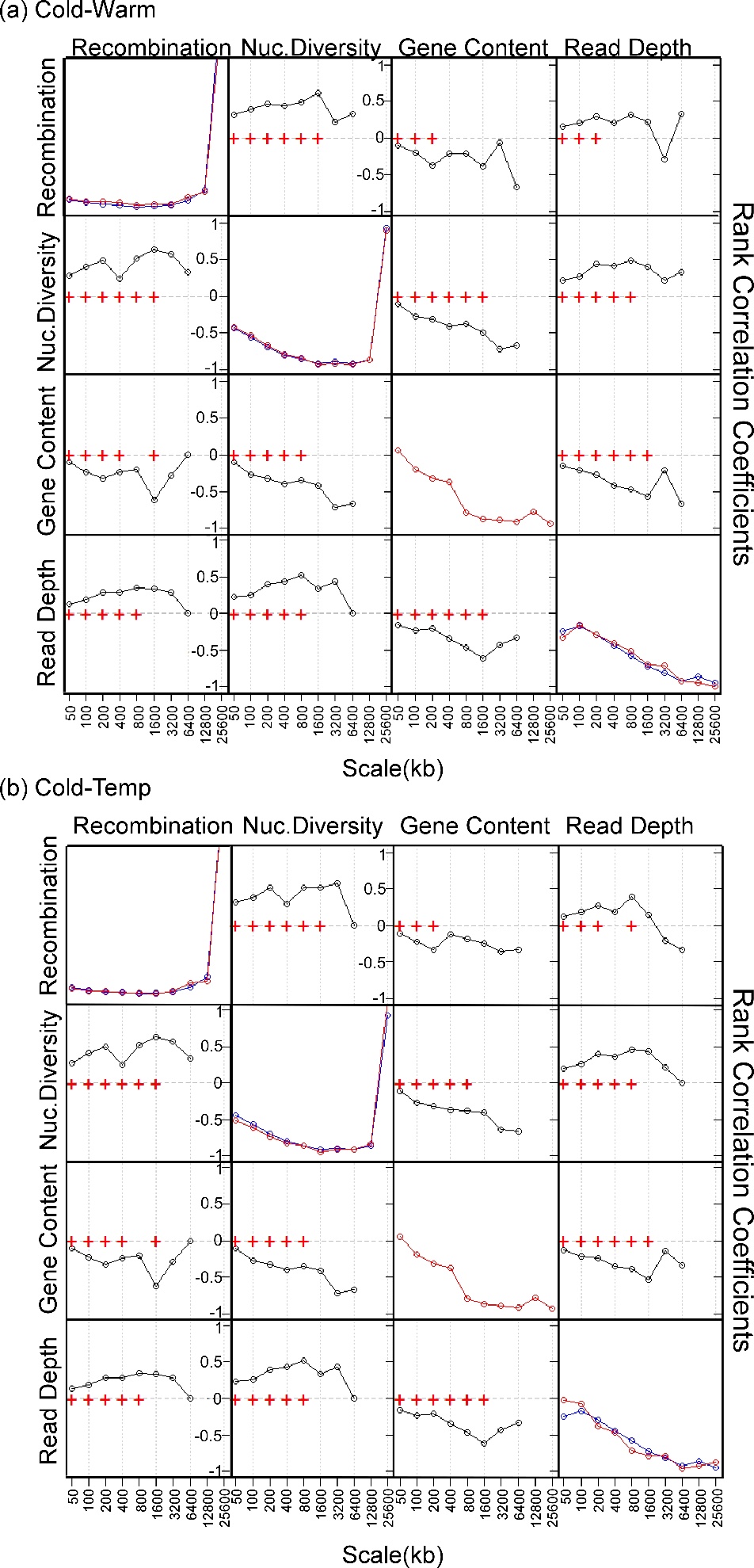


Supplementary Figure S6. Power spectra and pairwise rank correlation coefficients between detail wavelet coefficients, derived from the recombination maps for the three populations (Cold, Warm, and Temp) and the indicated genomic features for chromosome 3L. Off-diagonal plots indicate rank correlation coefficients between detail wavelet coefficients, derived from the population-specific recombination maps, and the genomic features. Red crosses denote correlations that are significant at the one percent level (Kendall’s rank correlation). To utilize even comparisons, the Cold population is utilized in both (a) and (b) and represents the matrix plot left and bottom of the diagonal with the Warm population (a) or Temp population (b) right and above the diagonal. Diagonal plots denote the wavelet power spectra of each indicated feature with the Cold population in blue and the Warm population (a) or Temp population (b) indicated in red.


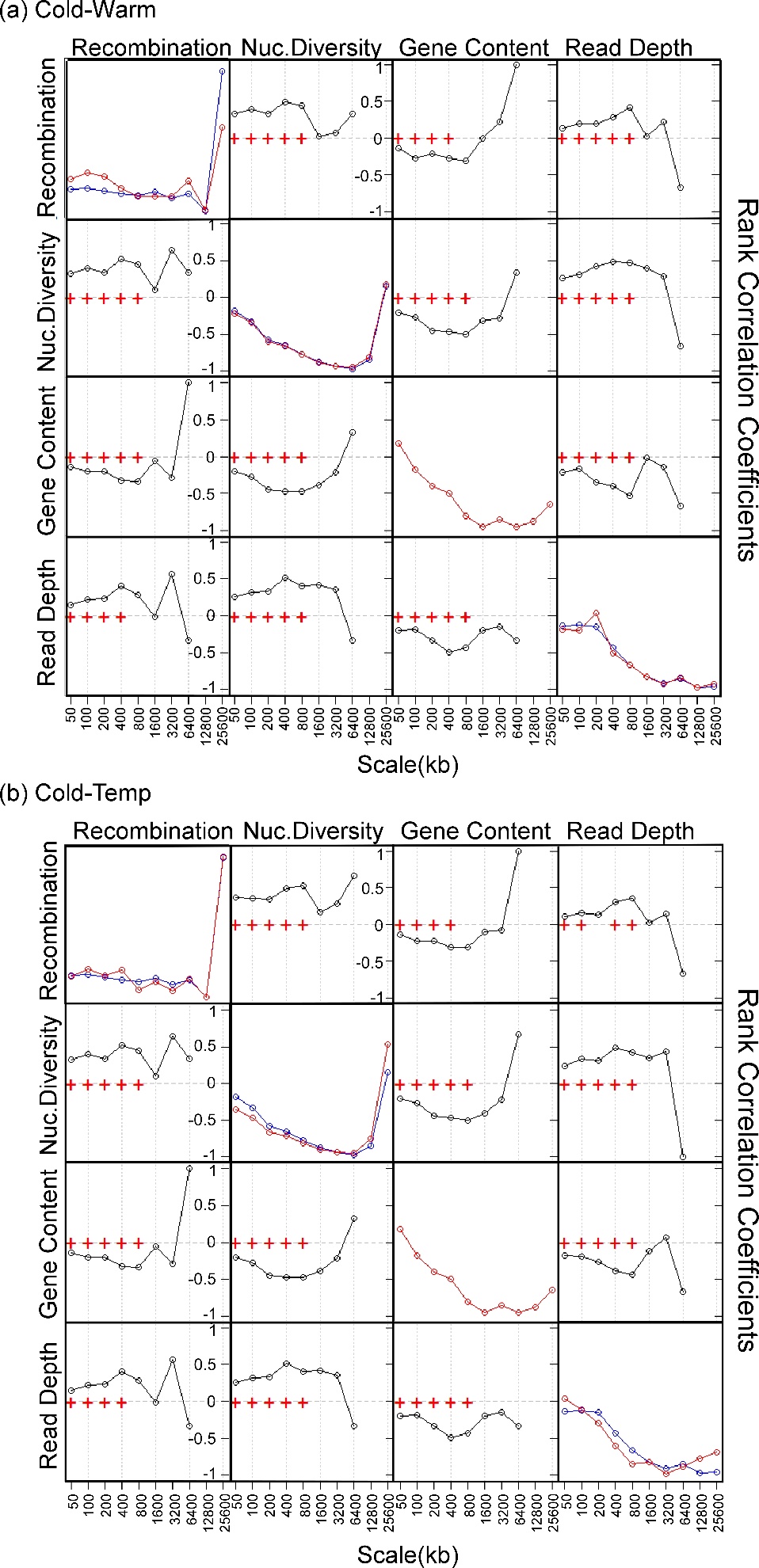


Supplementary Figure S7. Power spectra and pairwise rank correlation coefficients between detail wavelet coefficients, derived from the recombination maps for the three populations (Cold, Warm, and Temp) and the indicated genomic features for chromosome 3R. Off-diagonal plots indicate rank correlation coefficients between detail wavelet coefficients, derived from the population-specific recombination maps, and the genomic features. Red crosses denote correlations that are significant at the one percent level (Kendall’s rank correlation). To utilize even comparisons, the Cold population is utilized in both (a) and (b) and represents the matrix plot left and bottom of the diagonal with the Warm population (a) or Temp population (b) right and above the diagonal. Diagonal plots denote the wavelet power spectra of each indicated feature with the Cold population in blue and the Warm population (a) or Temp population (b) indicated in red.


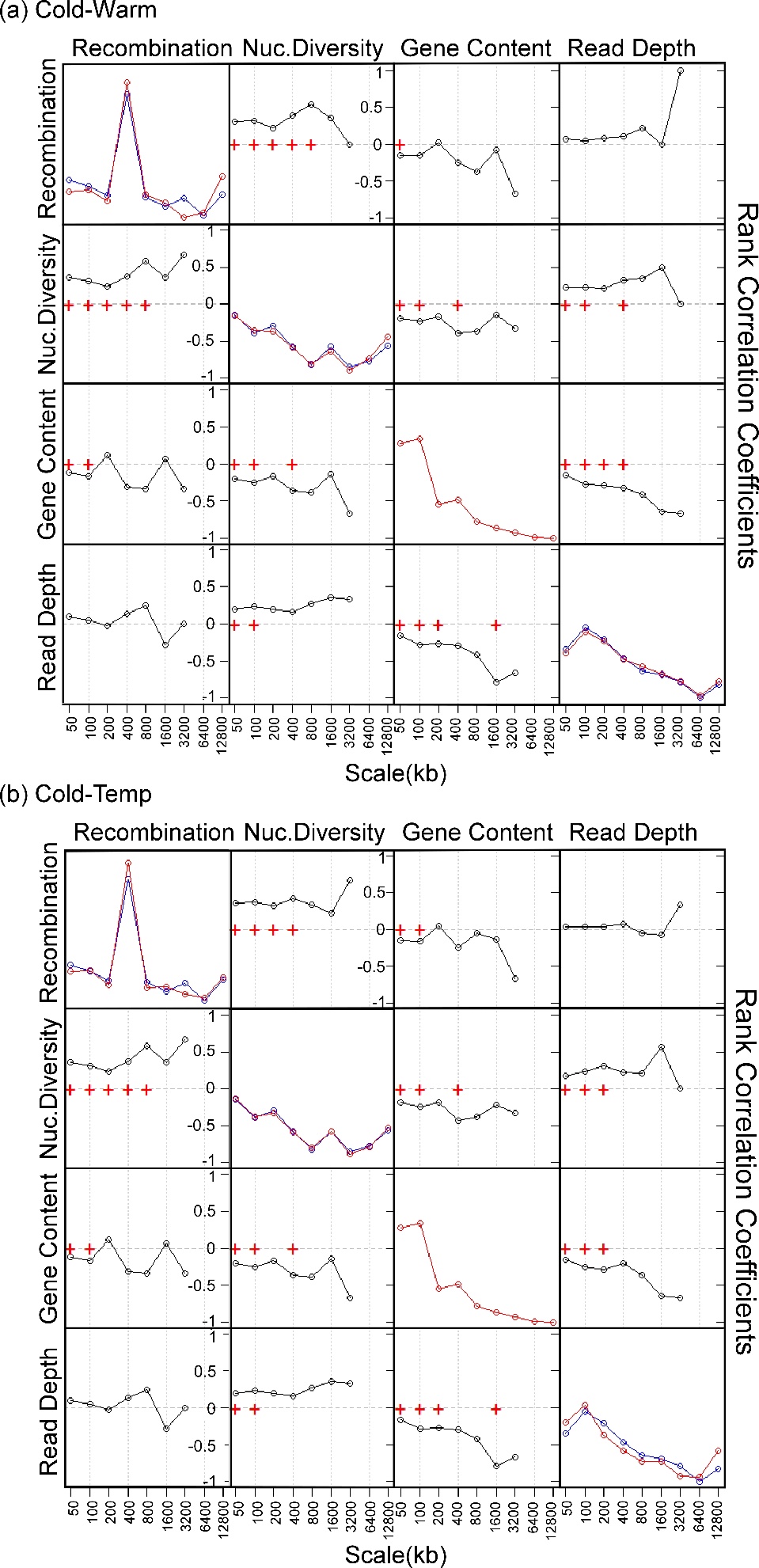


Supplementary Figure S8. Power spectra and pairwise rank correlation coefficients between detail wavelet coefficients, derived from the recombination maps for the three populations (Cold, Warm, and Temp) and the indicated genomic features for chromosome X. Off-diagonal plots indicate rank correlation coefficients between detail wavelet coefficients, derived from the population-specific recombination maps, and the genomic features. Red crosses denote correlations that are significant at the one percent level (Kendall’s rank correlation). To utilize even comparisons, the Cold population is utilized in both (a) and (b) and represents the matrix plot left and bottom of the diagonal with the Warm population (a) or Temp population (b) right and above the diagonal. Diagonal plots denote the wavelet power spectra of each indicated feature with the Cold population in blue and the Warm population (a) or Temp population (b) indicated in red.


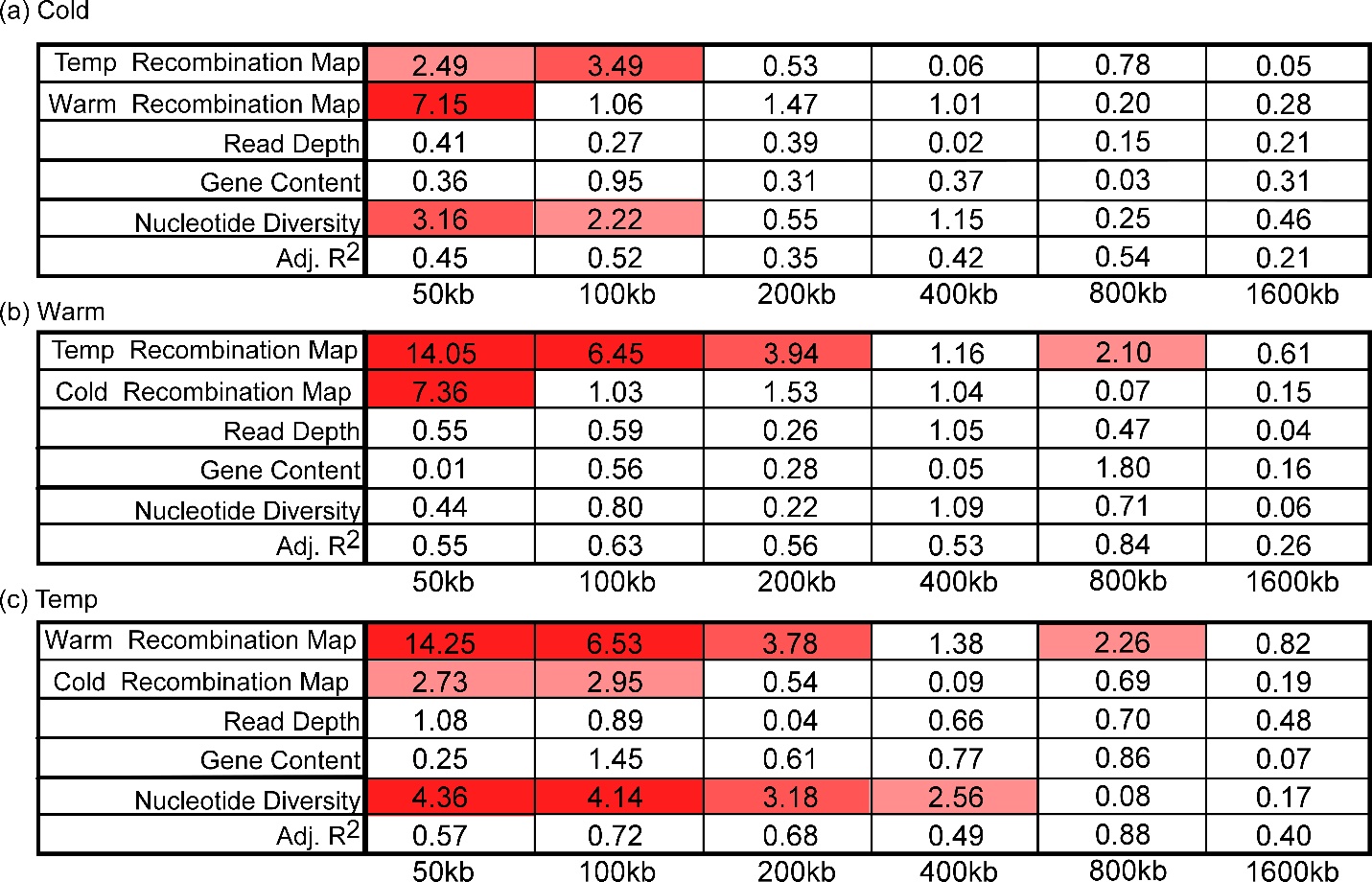


Supplementary Figure S9. Linear model for the detail coefficients of the wavelet transforms of the population specific recombination maps for the (a) Cold, (b) Warm and (c) Temp populations with the detail coefficients of the wavelet transforms of the indicated features serving as covariates/predictors in chromosome 2R. Values represent the -log_10_ P-value of the regression coefficient (t-test) and the adjusted r^2^ is included in the bottom row. Red/Blue boxes indicate a significant positive/negative linear relationship between the covariate and the recombination map at that scale. Note that for each population the other two remaining populations are also included as covariates/predictors.


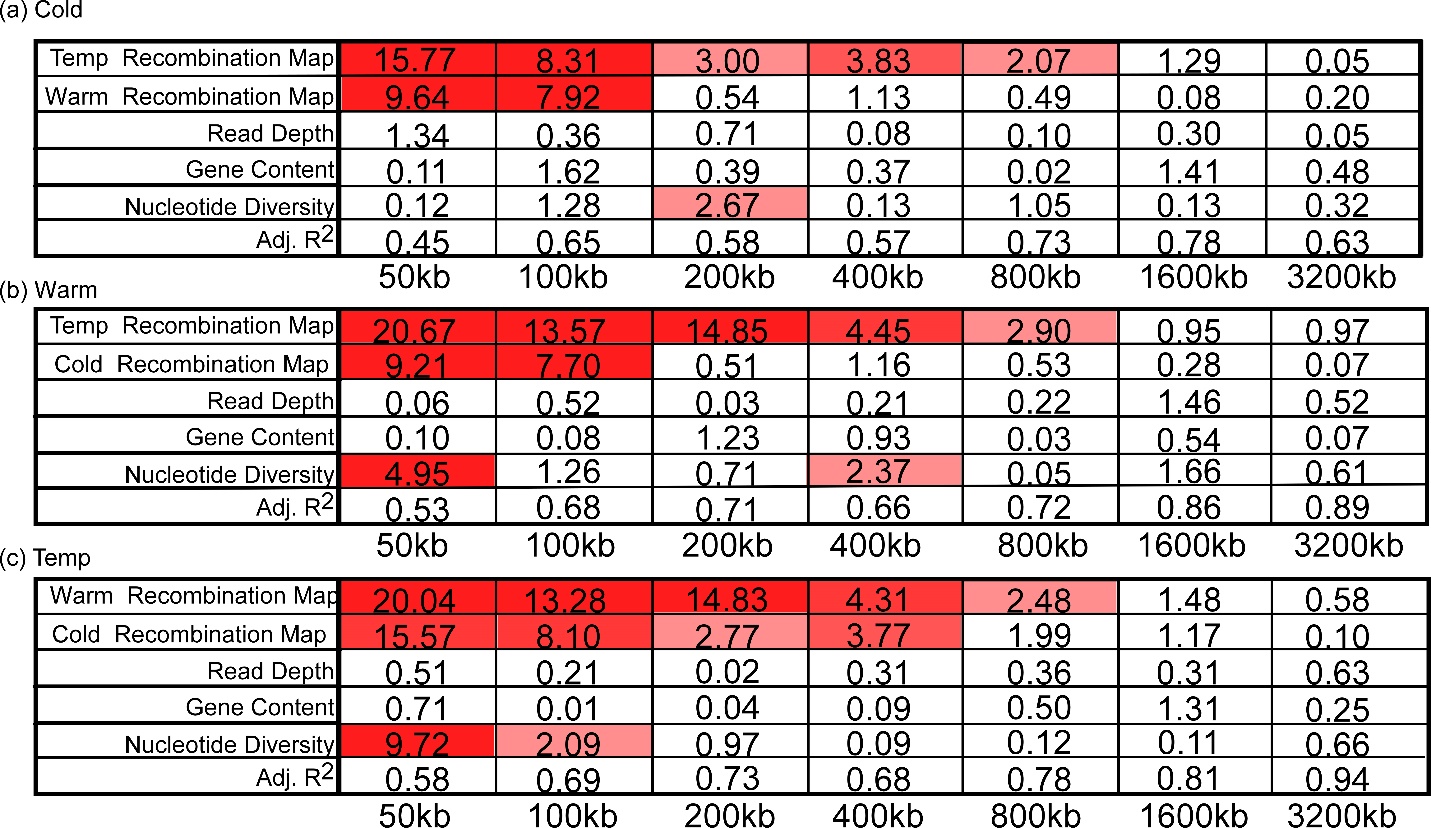


Supplementary Figure S10. Linear model for the detail coefficients of the wavelet transforms of the population specific recombination maps for the (a) Cold, (b) Warm and (c) Temp populations with the detail coefficients of the wavelet transforms of the indicated features serving as covariates/predictors in chromosome 3L. Values represent the -log_10_ P-value of the regression coefficient (t-test) and the adjusted r^2^ is included in the bottom row. Red/Blue boxes indicate a significant positive/negative linear relationship between the covariate and the recombination map at that scale. Note that for each population the other two remaining populations are also included as covariates/predictors.


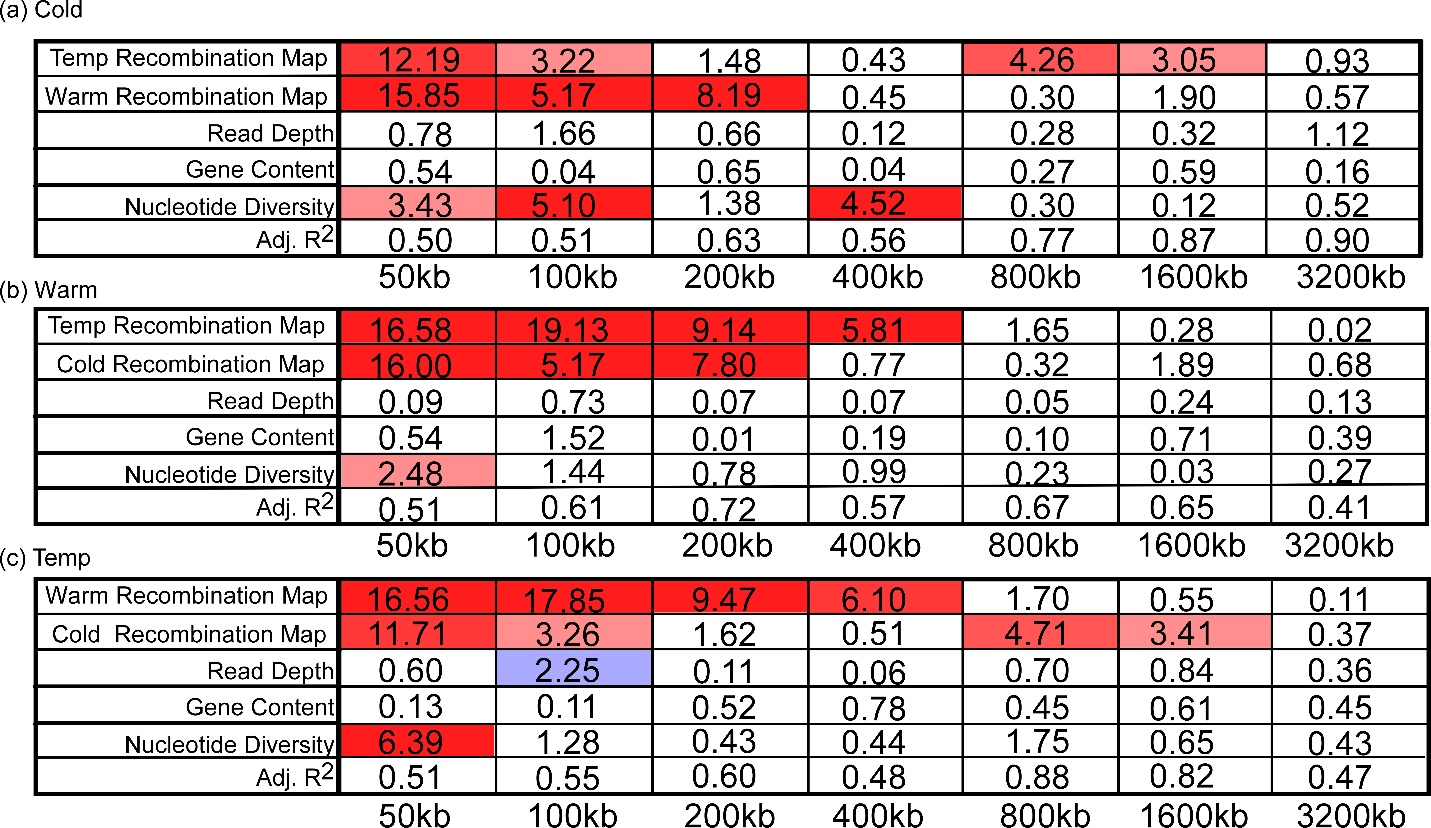


Supplementary Figure S11. Linear model for the detail coefficients of the wavelet transforms of the population specific recombination maps for the (a) Cold, (b) Warm and (c) Temp populations with the detail coefficients of the wavelet transforms of the indicated features serving as covariates/predictors in chromosome 3R. Values represent the -log_10_ P-value of the regression coefficient (t-test) and the adjusted r^2^ is included in the bottom row. Red/Blue boxes indicate a significant positive/negative linear relationship between the covariate and the recombination map at that scale. Note that for each population the other two remaining populations are also included as covariates/predictors.


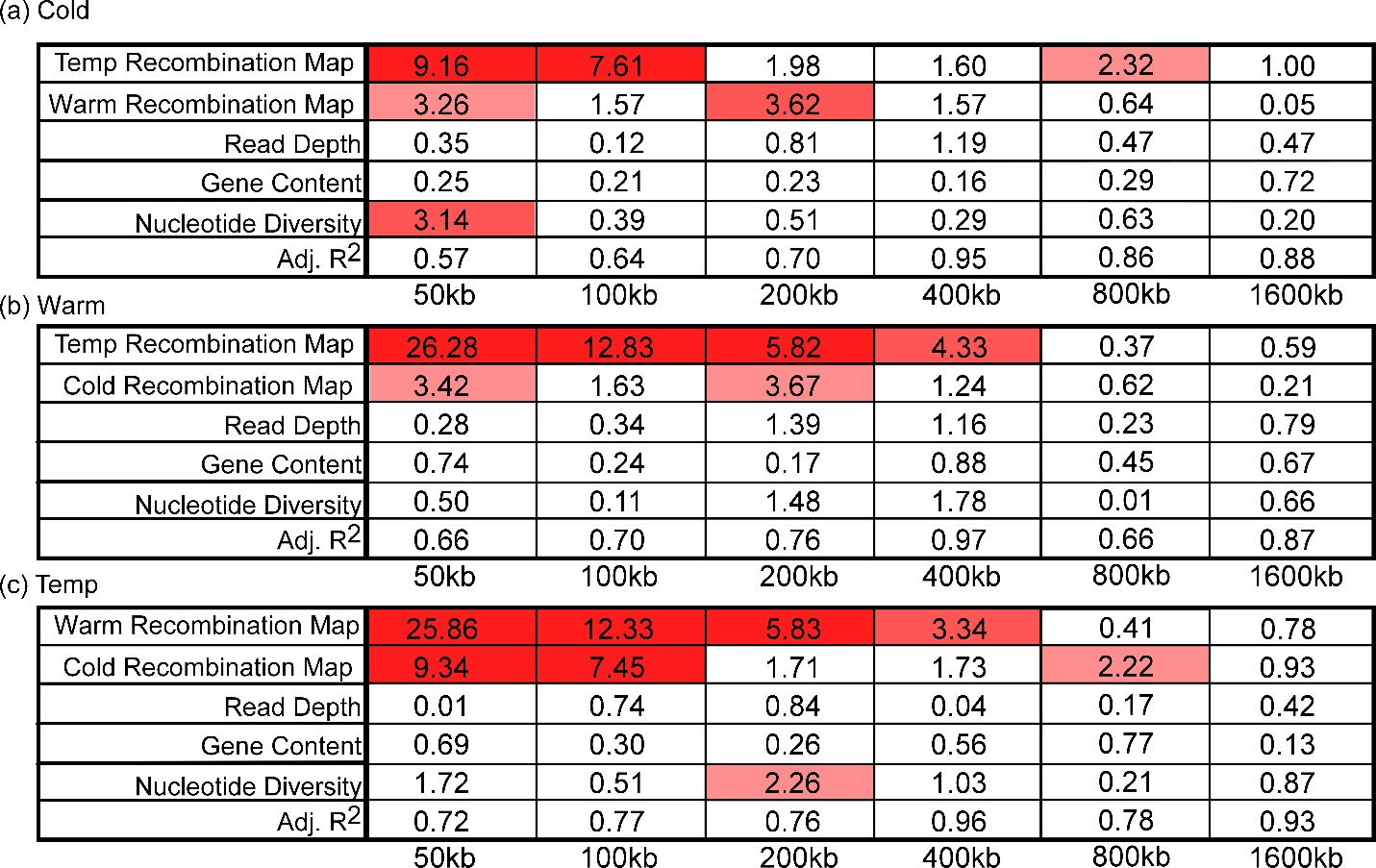


Supplementary Figure S12. Linear model for the detail coefficients of the wavelet transforms of the population specific recombination maps for the (a) Cold, (b) Warm and (c) Temp populations with the detail coefficients of the wavelet transforms of the indicated features serving as covariates/predictors in chromosome X. Values represent the -log_10_ P-value of the regression coefficient (t-test) and the adjusted r^2^ is included in the bottom row. Red/Blue boxes indicate a significant positive/negative linear relationship between the covariate and the recombination map at that scale. Note that for each population the other two remaining populations are also included as covariates/predictors.
